# ^18^F-FDG PET molecular imaging: A relevant tool to investigate chronic inflammatory rheumatisms in clinical practice?

**DOI:** 10.3389/fmed.2022.1070445

**Published:** 2022-11-30

**Authors:** Marie Pean De Ponfilly – Sotier, Raphaële Seror, Gaetane Nocturne, Florent L. Besson

**Affiliations:** ^1^Rheumatology, AP-HP. Université Paris-Saclay, Hôpital Bicêtre, Le Kremlin-Bicêtre, France; ^2^Faculté de Médecine, Université Paris-Saclay, Le Kremlin-Bicêtre, France; ^3^Université Paris-Saclay, INSERM, CEA, Centre de Recherche en Immunologie des Infections Virales et des Maladies Auto-immunes, Le Kremlin-Bicêtre, France; ^4^Biophysics and Nuclear Medicine-Molecular Imaging, AP-HP. Université Paris-Saclay, Hôpital Bicêtre, Le Kremlin-Bicêtre, France; ^5^Université Paris-Saclay, CEA, CNRS, Inserm, BioMaps, Orsay, France

**Keywords:** 18F-FDG, PET, rheumatoid arthritis, spondylarthropathy, polymyalgia rheumatica

## Abstract

^18^F-Labeled Fluorodeoxyglucose-Positron Emission Tomography (^18^F-FDG PET) is a molecular imaging tool commonly used in practice for the assessment of many cancers. Thanks to its properties, its use has been progressively extended to numerous inflammatory conditions, including chronic inflammatory rheumatism (CIR) such as rheumatoid arthritis (RA), spondylarthritis (SpAs) and polymyalgia rheumatica (PMR). ^18^F-FDG PET is currently not recommended for the diagnostic of CIRs. However, this whole-body imaging tool has emerged in clinical practice, providing a general overview of systemic involvement occurring in CIRs. Numerous studies have highlighted the capacity of ^18^F-FDG PET to detect articular and extra articular involvements in RA and PMR. However, the lack of specificity of ^18^F-FDG limits its use for diagnosis purpose. Finally, the key question is the definition of the best way to integrate this whole-body imaging tool in the patient’s management workflow.

## Introduction

**FIGURE 1:**
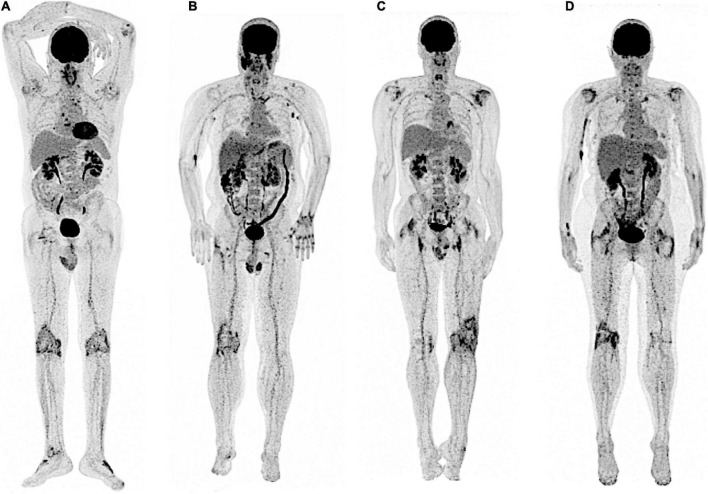
SpA and PMR: typical but non-specific periarticular patterns of ^18^F-FDG uptake at the individual whole-body level. In both SpA **(A,B)** and PMR **(C,D)**, typical increase of ^18^F-FDG uptake is frequently observed in the scapular and pelvic girdles as illustrated here, but also at sterno-clavicular joints and interspinous processes. In both CIR, peripheric articular involvement (knees) may also be observed. Typical ^18^F-FDG PET findings are thus non-specific at the patient level.

Positron Emission Tomography (PET) is a whole-body, non-invasive, and highly sensitive imaging modality based on the detection of radiolabeled vectors of interest. During the last 20 years, PET using ^18^F-fluoro-deoxy-glucose (^18^F-FDG), an analog of glucose radiolabeled with Fluor 18 (^18^F), has become a key imaging tool to diagnose, stage, and monitor many cancers in practice. Exploiting the metabolic properties of activated cells, the use of ^18^F-FDG has been progressively extended to numerous inflammation and infection disorders (1). A recent report from the European league Against Rheumatisms (EULAR) highlighted the heterogeneity of the availability to PET imaging for rheumatologic purpose across 25 European countries (2), which is currently dominated for the diagnosis of large vessel vasculitis (LVV) (3, 4) or to investigate fever of unknown origin (5). EULAR has provided official recommendations for the use of imaging in LVV, rheumatoid arthritis (RA) and spondylarthritis (SpAs) in clinical practice (2, 3, 6). While the use of PET modality is clearly defined only for LVV (3), ^18^F-FDG PET applied to chronic inflammatory rheumatisms (CIR) has concretely emerged in clinical practice (7, 8) (Figure 1 and Table 1). In this context, the choice of the best imaging modalities in patients with suspected CIRs – and the place of PET-CT in this strategy - arises at several stages of the management: to establish a positive diagnosis, to eliminate a differential diagnosis and in particular an infectious or para-neoplastic cause and finally to monitor the response to treatment.

**TABLE 1:** Characteristics of ^18^F-FDG PET-CT in RA, SpAs, and PMR.

	Rheumatoid arthritis	Spondylarthritis	Polymyalgia rheumatica
Positive diagnosis	^18^F-FDG uptake on peripheral joint (shoulder, elbow, wrist, hip, knee, ankle)	Specific but rare ^18^F-FDG uptake on sacro iliitis.	Bilateral and symmetric ^18^F-FDG uptake on gleno humeral, great trochanter, ischial tuberosities, sterno-clavicular joint and spinous process Similar FDG uptake for PMR-like irAEs
Differential diagnosis	Good discriminative performance for other RMDs;	Good discriminative performance for malignancies, infectious and mechanical back pain. Similarities between SpA and PMR requiring a composite score to discriminate both entities.	Good discriminative performance for certains RMDs, malignancies, paraneoplastic PMR-like syndrome and infectious. Similarities between SpA and PMR requiring a composite score to discriminate both entities.
Associated diseases	• Inflammation of the aortic wall Possibly correlated with CVD risk factors. Non-clear effect of the treatment and disease activity on the outcome of the inflammation. • Detection of subclinical myocardial inflammation Correlated with disease activity		LVV: • Official recommendation for suspicion of GCA isolated or associated with PMR • Good performance for the detection of subclinical GCA in patients with isolated symptoms of PMR
Treatment outcome	Good correlation between ^18^F-FDG PET-CT and disease activity (DAS28, acute phase reactant, patient’s global assessment, ultrasound)	One study: No correlation between clinical report disease activity (BASDAI, BASFI) and ^18^F-FDG PET-CT. Require more data.	Not relevant neither for monitoring treatment response nor disease activity.
Other tracers	Zirconium-89 (B-cell target) [11C](R)PK11195 (macrophages target)	^18^F-Na, ^18^F-fluoride PET (osteoblastic activity)	

RMD, Rheumatic Musculo-skeletal diseases; AS, ankylosing spondylarthritis; BASDAI, Bath Ankylosing Spondylitis Disease Activity Index; BASFI, Bath ankylosing spondylitis functional index; irAEs, immune related adverses events.

The aim of this short review is to summarize the evidence-based literature on the benefits of ^18^F-FDG PET imaging in patients with CIR, focusing on rheumatoid arthritis (RA), spondyloarthritis (SpA) and polymyalgia rheumatica (PMR). Promising other radiotracers of interest in this field will also be discussed.

### ^18^F-FDG PET in rheumatoid arthritis

Rheumatoid arthritis is the most frequent CIR affecting 0.3–0.5% of the general population (9, 10). RA is characterized by an inflammation of synovial membrane (synovitis) resulting in bone erosion. The frequency of systemic manifestations seems to be decreasing apart from pulmonary involvement which affects about 20% of patients (11). According to the ACR/EULAR 2010 classification criteria (12), the diagnosis is based on clinical presentation and biological criteria [Acute-phase reactants, anti – cyclic citrullinated peptide2 antibodies (ACPA2) and/or rheumatoid factor (RF)]. Imaging by X-ray is required to look for structural damage (joint erosions).

Although ^18^F-FDG PET is not currently validated in this case, PET molecular imaging has demonstrated its diagnostic value in atypical and challenging cases. Bhattarai et al. (13) reported good performance of ^18^F-FDG PET combined with computed tomography (PET-CT) to discriminate 18 RA from 17 non-RA patients (SAPHO syndrome, IgG4 arthritis, psoriatic arthritis and non-specific arthritis). Compared to non-RA patients, RA patients had significantly higher metabolic visual score defined by the sum of the maximum standardized uptake value of each joint (shoulders, elbow, wrist, hip, knee, and ankle). In the same way, Yamashita et al. (14) demonstrated significant differences of ^18^F-FDG uptake on ischial tuberosity, great trochanter, spinous process, vertebral body and sacroiliac joint between RA, PMR, and SpA patients. Recently, Wang et al. (15) demonstrated that ^18^F-FDG PET could discriminate 54 suspected RA (*n* = 23) from PMR (*n* = 31) patient, based on interspinous uptake combined with rheumatoid factor (AUC = 0.892).

Concerning the assessment of disease activity, Lee et al. investigated 91 active and mainly treatment-naïve RA with a mean Disease activity score of 28, C – Reactive Protein (DAS28 CRP) of 6.4. While univariate analyses showed correlations between PET-positive joint and clinic-biological symptoms (the swollen joint and Tender Joint Counts, DAS28 CRP and Erythrocyte sedimentation rate), multivariate analyses confirmed a positive correlation between PET positive joint and DAS28 ESR and patient’s global disease score. Beckers et al. (16) supported these result and further demonstrated relationship between ^18^F-FDG PET and ultrasonography to detect inflamed joints. However, the use of PET-CT for this purpose of monitoring response to therapy in RA patients is not sound in clinical practice. The gold standard should be clinico-biological follow-up by DAS28 score and structural follow-up by X-ray.

As a prognosis marker in RA, few ^18^F-FDG PET studies have shown positive results. Roivainen et al. (17) have assessed the early predictive value of ^18^F-FDG PET in RA patients treated by Methotrexate/Salazopyrine/Hydroxychloroquine tri-therapy. In 17 RA patients, ^18^F-FDG-PET was performed at baseline, 2 and 4 weeks after the initiation of treatment. Interestingly, a decrease of disease activity on PET after 2 and 4 weeks of treatment correlated with DAS28 and CRP at 12 weeks. Elzinga et al. (18) supported these preliminary results with Infliximab, a monoclonal inhibitor of TNF, by showing a correlation between the early PET changes at 2 weeks and DAS28 at 14 and 22 weeks. Same performance was also reported with Tocilizumab, an interleukine – 6 inhibitor (19). To note, Bouman et al. did not supported this prognosis value in RA patients with low disease activity treated by TNF inhibitors (20). Again, the clinical usefulness arises. It would be useful to evaluate the economic cost and benefit-risk balance of performing 2 PET scans a few weeks apart in patients versus conventional clinical-biological monitoring.

Finally, the holistic nature of ^18^F-FDG as a biomarker of cellular metabolic activity makes ^18^F-FDG PET an intrinsic “swiss-knife” to evaluate multidimensional aspects of the same disease, even at a subclinical level. Due to vascular wall and/or systemic chronic inflammation condition, RA patients had an increased risk of cardiovascular diseases (CVD) (21, 22), up to 2–3-fold excess of mortality in comparison with general population (23). In their recent study, Geraldino-Pardilla et al. (22) showed in 91 RA patients without clinical evidence of CVD a significant relationship between ^18^F-FDG PET vascular uptake of the aortic wall and both CVD risk factors (arterial hypertension and body mass index) and RA disease features (rheumatoid nodules and Disease activity score) (22). In the same way, Trang et al. (24) showed in 64 RA patients increased ^18^F-FDG uptake of the aortic wall after 6 months of biologic therapies (TNF inhibitors, IL6 blockers and Ig CTLA4), even in RA patients with low disease activity or in clinical remission. Moreover, Amigues et al. reported subclinical myocardial ^18^F-FDG uptake in 39% of their 119 RA patients without known CVD (25). For 8 patients requiring an step-up of their treatment, the longitudinal follow-up showed substantial decrease of myocardial ^18^F-FDG uptake over 6 months, together with the clinical disease activity index. In a controlled study including 33 RA and age/gender matched controls with neither RA nor CVD, a significant correlation between synovial (acromioclavicular and acetabulo-femoral joints) and aortic ^18^F-FDG uptake was observed only in the RA group (26). These results are interesting. Nevertheless, it would be necessary to define the pathological value of vascular abnormalities in RA patients and to be able to determine whether their detection should result in therapeutic intervention.

In summary, there are abnormalities specific to RA on PET CT. However, it is not clear whether this examination has a place in the diagnostic or follow-up strategy of patients.

### ^18^F-FDG PET in spondylarthritis

Spondylarthritis is a composite spectrum of rheumatism disorders sharing common clinical and genetic features, including ankylosing spondylitis (AS), reactional arthritis, arthritis associated to bowel inflammatory diseases, psoriatic arthritis, undifferentiated SpA and Synovitis – Arthritis – Palmoplantar pustulosis - Hyperostosis – Osteitis syndrome (SAPHO). SpA affects 0.2 to 0.3% of the general population and typically concerns young males below 40 years old. Musculoskeletal manifestations include pelvic and axial inflammatory pain, peripheral joint involvement and enthesis (27). SpA had no specific biologic marker contrary to RA. Conventional radiography and ultrasonography typically lack of sensitivity (28). Magnetic resonance imaging (MRI) remains the gold standard to assess spine and sacroiliac joint (SIJ), SIJ involvement being critical according to the diagnostic criteria of the 2009 Assessment of Spondyloarthritis international Society classification (29).

**FIGURE 2:**
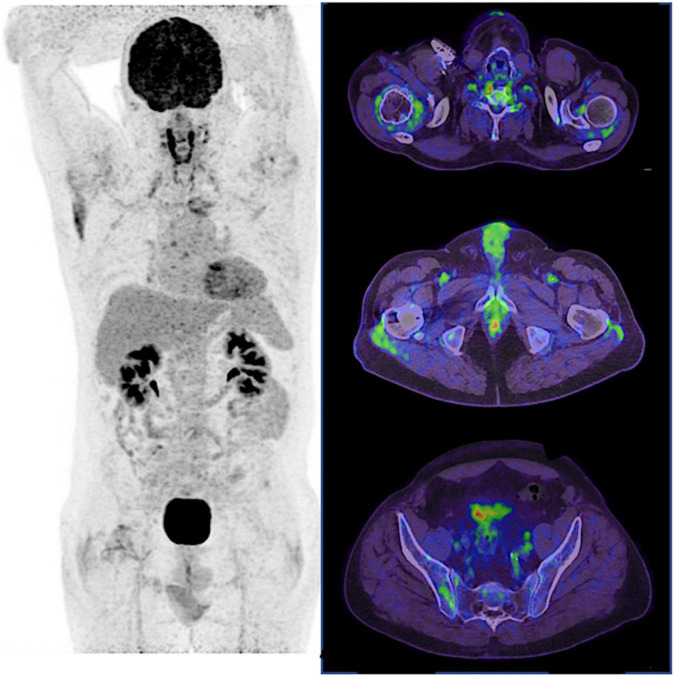
^18^F-FDG PET of SpA. In SpA, sacro-iliitis is a highly specific pattern of ^18^F-FDG uptake, as illustrated here in the right sacro-iliac joint, but is rarely observed in practice. As for PMR, ^18^F-FDG uptake of the sterno-clavicular joints, scapular and pelvic girdles is also observed.

Despite the lack of clinical validation, ^18^F-FDG PET-CT showed interesting results as a diagnostic tool in SpA (Figure 2). In a challenging clinical context mixing 21 SpA, 16 RA and 16 PMR patients, Yamashita et al. found higher ^18^F-FDG uptake in sacroiliac joint of SpA patients compared to other CIR (14). To note, only 60% of the sacroiliitis diagnosed with MRI were identified on PET-CT, and no inter-groups difference of ^18^F-FDG uptake was observed for the other joints (ischiatic tuberosity, greater trochanter, spinous process, and vertebral body). These data are supported by Strobel et al. (30) who found moderate diagnostic performance of ^18^F-FDG PET-CT to detect sacroiliitis in 28 patients with active AS (*n* = 15) or mechanical low back pain (MLBP, n = 13), especially for grades II (localized erosions or scleroses, no alteration in the joint width, Se = 40%) and IV (ankylosis, Sen = 50%). Recently, Pean de Ponfilly-Sotier et al. (31) evaluated ^18^F-FDG PET-CT in a particular population of 27 atypical SpA mixing late onset SpA patients, patients refractory to TNF inhibitors and/or with general manifestations. In this specific challenging population, ^18^F-FDG PET-CT showed rare but higher ^18^F-FDG uptake in SIJ compared to PMR patients. Other ^18^F-FDG uptake locations (ischial tuberosity, great trochanter, hips, shoulders, interspinous process) were significantly associated with PMR. At the patient level, ^18^F-FDG PET could not discriminate PMR from atypical SpA (Figure 1). In another study by Toussirot et al. (32), ^18^F-FDG PET-CT demonstrated a good concordance with MRI to detect sacroiliitis and spinal inflammatory lesions in AS. However, in non-radiographic axial SpA patient, ^18^F-FDG-PET-CT showed no metabolic activity and did not seem to be helpful in this specific population, suggesting ^18^F-FDG PET-CT to be relevant in patients with active SpA. In summary, PET CT may have value in the diagnostic approach to atypical presentations in patients with suspected SpA.

Monitoring the disease independently from the self-reported clinical score Bath Ankylosing Spondylitis Disease Activity Index (BASDAI) and Bath ankylosing spondylitis functional index (BASFI) remains an important issue in SpA. In this perspective, Wendling et al. evaluated three AS patients with ^18^F-FDG PET-CT at baseline and after 6–8 weeks of TNF inhibitors (33). No decrease in ^18^F-FDG uptake was observed under treatment, highlighting the need of further large cohort investigations in this particular field.

### ^18^F-FDG PET in polymyalgia rheumatica

**FIGURE 3:**
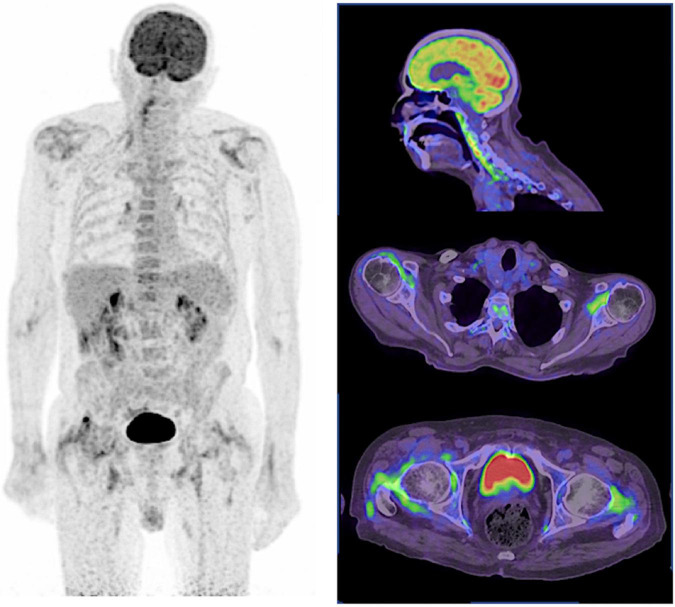
^18^F-FDG PET of PMR. In PMR, active LVV frequently overlaps, as illustrated here with the long linear and smooth 18F-FDG uptake of the right vertebral artery in this corticosteroid resistant PMR patient. As for SpA, ^18^F-FDG uptake of the sterno-clavicular joints, scapular, and pelvic girdles is also typically observed.

Polymyalgia rheumatic (PMR) is an inflammatory disease of unknown origin affecting patients over 50 years old, causing arthromyalgia of the pelvic and/or scapular girdle, systemic manifestations and sometimes peripheral arthritis (34, 35). A biologic inflammatory syndrome is classical. As well as SpA, PMR has no specific biomarkers. The diagnosis is based on clinical and laboratory evidence and the exclusion of the numerous differential diagnosis (e.g., late onset SpA, rheumatoid arthritis with rhizomelic presentation, remittive symmetrical seronegative synovitis with pitting edema (RS3PE), inflammatory myositis ….) with plain radiography and exhaustive biologic tests. In 15 to 20% of cases, PMR is associated with giant cell arteritis (GCA), an inflammatory disease of the vascular wall of large arteries (Figure 3). Corticosteroids are the cornerstone of treatment both for GCA and PMR.

Although ^18^F-FDG-PET-CT is not recommended to diagnose isolated PMR, ^18^F-FDG PET-CT is now indicated as a first line imaging procedure in the case of suspected GCA (3). Historically, Blockmans et al. were the first to apply ^18^F-FDG PET on isolated PMR patients (36). In their seminal paper, the authors reported the currently well-known reference pattern of ^18^F-FDG uptake in this RIC: a bilateral and symmetrical increase of ^18^F-FDG uptake in the shoulders and pelvic girdles, frequently associated with multi-tiered increase of ^18^F-FDG uptake of spinous process. During the last 15 years, several studies reported these typical ^18^F-FDG uptake, but also sternoclavicular joints (14, 31, 37). At the population level, the intensity of ^18^F-FDG uptake in these locations appears higher in PMR patients compared to RA, SpA and other inflammatory condition (38). A recent meta-analysis by van der Geest et al. showed that combining these targeted anatomic sites of ^18^F-FDG uptake improved the diagnosis performance of PET in PMR (37). These data were later confirmed by other studies (31, 39), highlighting the fact that ^18^F-FDG-PET-CT could be a relevant tool to discriminate PMR from other inflammatory rheumatisms, even in challenging case-mix populations. Steroid treatment prior to PET-CT reduces the scan’s ability to demonstrate inflammation in PMR patients. This should be kept in mind when interpreting PET-CT in patients already exposed to steroids (40). PMR-like syndrome have been reported in patients receiving immune check point inhibitors (ICIs) for cancers (41, 42). Rheumatic and Musculo-skeletal immune-related adverse events (irAes) often do not fulfill to the traditional classification criteria (42). Compared with classical PMR, PMR-like syndromes showed higher prevalence of peripheral arthritis and the biologic inflammation can be lacking (43). Previous report (44) and van der Geest et al. (45) assessed the role of ^18^F-FDG PET-CT before the initiation of corticosteroid in 6 patients with PMR – like syndrome. He found the same symmetric ^18^F-FDG uptake locations than those in classical PMR, without LVV associated. One third exhibited peripheral ^18^F-FDG uptake. These results were confirmed recently by Ponce et al. (44). To note, patients experienced irAEs induced by ICI had a better cancer prognosis than those in non – irAEs patients (46).

**FIGURE 4:**
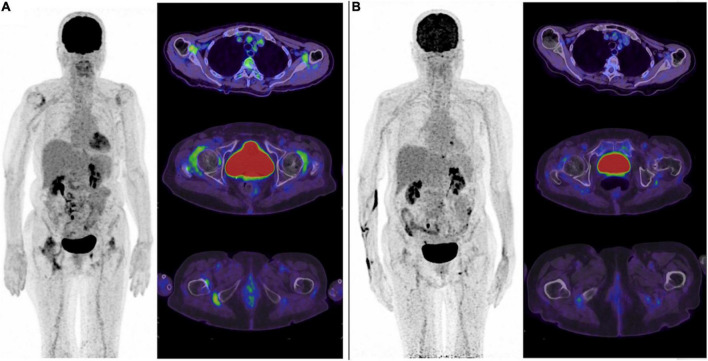
^18^F-FDG PET changes under treatment: typical but no current benefit over standard biomarkers. In this case of PMR, significant decrease of ^18^F-FDG uptake is observed in the targeted joints (here the scapular and pelvic girdles, ischial tuberosities) under treatment **(A)** baseline scan, and **(B)** after several lines of treatment). Although metabolic changes assessed with ^18^F-FDG are frequently observed, the clinical benefit of such imaging biomarker over standard clinical and biological biomarkers for disease monitoring remains to be defined.

Monitoring the response to treatment with ^18^F-FDG PET-CT may be tempting (Figure 4). However, standard clinical and biological biomarkers are currently sufficient in most practical cases. Nevertheless, Palard-Novello et al. assessed the value of ^18^F-FDG PET-CT to monitor 18 PMR patients receiving Tocilizumab as first line treatment (TENOR trial) (47). The PET-CT were performed at baseline, two and 12 weeks. Between baseline and 12 weeks, the authors observed significant improvement of the PMR activity score and biological markers, together with a decrease of ^18^F-FDG uptake in targeted joints (hips, ischial tuberosity, lumbar spinous process). During follow-up, no correlation was found between PET, clinical and biological biomarkers. Devauchelle-Pensec et al. confirmed these results in a cohort of 20 glucocorticoid-free PMR onset receiving Tocilizumab as first line treatment (48). Thus ^18^F-FDG PET-CT may not appear relevant neither for monitoring treatment response nor disease activity in PMR patients.

Whether ^18^F-FDG-PET-CT could be a relevant tool or not to predict response to treatment is poorly evaluated. In 2007, the seminal paper by Blockman et al. (36) found no difference of ^18^F-FDG uptake between relapsers and non-relapsers at 3 and 6 months, and ^18^F-FDG PET uptake was not correlated with the risk of relapse. In a recent study, Prieto-Peña et al. (49) identified predictive factor of ^18^F-FDG PET positivity for LVV in a population of 84 isolated PMR patient with persistent of classical PMR symptoms and/or unusual symptoms (inflammatory low back pain, diffuse lower limb pain). Among 84 patients, 51 (61%) patients had evidence of LVV on ^18^F-FDG PET. In multivariate analysis, diffuse lower limb pain, pelvic girdle pain and inflammatory low back pain were the best set of predictors of PET positivity for LVV in patients with initially isolated PMR. Finally, a take home message of this study is the potential existence of LVV signs in PMR patients in whom GCA was not clinically suspected. The presence of predictive signs could raise the question of systematically searching for GCA by PET CT given the therapeutic and prognostic impact. This work will therefore need to be confirmed in a new cohort. As mentioned previously, caution should be made in patients already exposed to glucocorticoids. Because glucocorticoids rapidly reduce the ^18^F-FDG uptake of the vascular wall in LVV patients, withdraw or delay therapy until after ^18^F-FDG PET when possible (i.e., no risk of ischemic complications) or ^18^F-FDG PET acquisition within the first days of therapy are currently recommended to reduce the risk of false negative results (4).

Finally, ^18^F-FDG PET could be used for differential diagnosis including cancers revealed by musculoskeletal manifestations including PMR-like symptoms. In this perspective, Moya-Alvarado et al. (50) assessed the added value of ^18^F-FDG PET-CT to diagnose other underlying conditions in a cohort of 103 onset and steroid resistant PMR patients. The final diagnosis of PMR, LVV, malignancies and other (small vessel vasculitis, osteoarthritis, elderly onset of RA, Sjögren’s syndrome) were retained in 73, 16, 5, and 9 patients, respectively. In the case of bio-clinical flare after glucocorticoid tapering in GCA patients with or without PMR, Camellino et al. promote the use of ^18^F-FDG PET-CT to rule-out cancer and detect subclinical LVV (35).

### Time for new tracers?

PET imaging is characterized by its vectorized imaging capabilities. Beyond ^18^F-FDG we have focused on here, other tracers have been investigated in CIRs (51). In SpA, several studies reported the relevance of ^18^F-Na (^18^F-fluoride PET) to evaluate osteoblastic activity in chronic AS, and diagnose new bone formation in spine and SIJ which were misjudged by MRI and delayed by plain radiography (52–54). Son et al. (55) evaluated ^18^F-Na in a retrospective cohort of 68 patients with suspected AS. Among 68 patients, 72% reach ASAS criteria for SpA. Eighty percent in AS group exhibited higher frequency of ^18^F-Na uptake (enthesopathy, syndesmophyte, symmetric sacroiliitis), in comparison with the control group. In 2018, in a pilot study, Bruijnen et al. (53) suggested that AS activity was better reflected by bone activity assessed by ^18^F-Na than inflammation assessed by ^18^F-FDG and [11C](R)PK11195, a radiotracer of inflammation targeting the mitochondrial outer membrane translocator protein of activated macrophages (TSPO PET). In RA, TSPO PET radiotracers have also been assessed (56, 57). In their study including 29 RA patients without clinical arthritis, Gent et al. showed increased TSPO PET uptake in metacarpophalangeal, proximal interphalangeal, and wrist joints in 55% of cases, of whom 69% developed a flare within the 3-years of follow-up (56). More recently, the baseline PET assessment of B-cell load by using radiolabeled Rituximab (Zirconium-89) showed independent value of PET to prognose therapeutic response, with positive and negative predictive values for clinical response at 24 weeks of 90 and 75%, respectively (58).

To conclude, the place of ^18^F-PET-CT in the management of patients with RA, SpA and PMR remains to be defined. In classical presentations, ^18^F-FDG PET does not appear useful in the diagnostic process. It should be reserved for atypical presentations or in case of poor response to treatment in order to ensure the absence of a differential diagnosis. Its relevance for the assessment of associated manifestations - cardiovascular complications of RA - GCA associated with PMR appears promising, deserving dedicated lines of research. Finally, the use of new tracers is to be followed to improve the diagnostic and prognostic performance of this whole-body imaging modality.

## Author contributions

All authors contributed to design and acquisition analysis, revising for intellectual content, final approval, and agreement to be accountable for all aspects of this work (accuracy and integrity of any part of the work).

## Abbreviations

^18^F-FDG PET-CT: ^18^F-labeled fluorodeoxyglucose-positron emission tomography/computed tomography
CIR: chronic inflammatory rheumatism
RA: rheumatoid arthritis
SpAs: spondylarthris
PMR: polymyalgia rheumatica
ACPA2: anti – cyclic citrullinated peptide2 antibodies
RF: rheumatoid factor
SUVmax: maximum standardized uptake value
DAS28 CRP: Disease activity score 28 C – Reactive Protein
SJC: Swollen joint count
TJC: Tender Joint Count
CVD: cardiovascular diseases
RLDA: Remission or low disease activity
MHDA: Moderate or high disease activity
AS: ankylosing spondylitis
SAPHO: Synovitis – Arthritis – Palmoplantar pustulosis - Hyperostosis – Osteitis syndrome
SIJ: sacroiliac joint
MLBP: mechanical low back pain
BASDAI: Bath Ankylosing Spondylitis Disease Activity Index
BASFI: Bath ankylosing spondylitis functional index
ROC: Receiver Operating Characteristic Curve
MRI: Magnetic resonance imaging
AUC: Area Under the Curve
SpA: Spondylarthritis
TNFi: TNF inhibitors
PMR: Polymyalgia rheumatic
GCA: giant cell arteritis
LVV: Live vessel vasculitis
RS3PE: remittive symmetrical seronegative synovitis with pitting edema
ICIs: immune check point inhibitors
irAes: immune-related adverse events
RMDs: Rheumatologic and Musculo-skeletal Disorders.
